# Ovarian cancer plasticity and epigenomics in the acquisition of a stem-like phenotype

**DOI:** 10.1186/1757-2215-1-8

**Published:** 2008-11-24

**Authors:** Nicholas B Berry, Sharmila A Bapat

**Affiliations:** 1 National Centre for Cell Science, NCCS Complex, Pune University Campus, Pune 411007, INDIA

## Abstract

Aggressive epithelial ovarian cancer (EOC) is genetically and epigenetically distinct from normal ovarian surface epithelial cells (OSE) and early neoplasia. Co-expression of epithelial and mesenchymal markers in EOC suggests an involvement of epithelial-mesenchymal transition (EMT) in cancer initiation and progression. This phenomenon is often associated with acquisition of a stem cell-like phenotype and chemoresistance that correlate with the specific gene expression patterns accompanying transformation, revealing a plasticity of the ovarian cancer cell genome during disease progression.

Differential gene expressions between normal and transformed cells reflect the varying mechanisms of regulation including genetic changes like rearrangements within the genome, as well as epigenetic changes such as global genomic hypomethylation with localized promoter CpG island hypermethylation. The similarity of gene expression between ovarian cancer cells and the stem-like ovarian cancer initiating cells (OCIC) are surprisingly also correlated with epigenetic mechanisms of gene regulation in normal stem cells. Both normal and cancer stem cells maintain genetic flexibility by co-placement of activating and/or repressive epigenetic modifications on histone H3. The co-occupancy of such opposing histone marks is believed to maintain gene flexibility and such bivalent histones have been described as being poised for transcriptional activation or epigenetic silencing. The involvement of both-microRNA (miRNA) mediated epigenetic regulation, as well as epigenetic-induced changes in miRNA expression further highlight an additional complexity in cancer stem cell epigenomics.

Recent advances in array-based whole-genome/epigenome analyses will continue to further unravel the genomes and epigenomes of cancer and cancer stem cells. In order to illuminate phenotypic signatures that delineate ovarian cancer from their associated cancer stem cells, a priority must lie in the expansion of current technologies and further implementation of bioinformatics to handle the complexity of the cancer epigenome and the various networks that coordinate disease initiation and progression. Great potential lies in the translation of these findings into epigenetic-based therapies. Additionally, targeting chemo-resistant cancer stem cells may provide a much needed breakthrough in treatment of advanced ovarian cancer and chemoresistant disease.

## Background

Epithelial ovarian cancer (EOC) is the eighth most common cancer among women and causes more deaths than any other female reproductive tract cancer [1]. The American Cancer Society estimates that about 21,650 new cases of ovarian cancer will be diagnosed in the United States during 2008. A woman's risk of getting invasive ovarian cancer during her lifetime is about 1 in 71, and her lifetime chance of dying from invasive ovarian cancer is about 1 in 95 [2]. Ovarian cancer strikes silently, usually revealing no obvious symptoms until disease advances to a metastatic stage. The standard treatment is cytoreductive surgery followed by platinum/taxane regimens, which results in clinically complete remissions in > 70% of patients. However, relapse occurs in > 90% of those responders, at which point the disease is essentially incurable. Drug resistance remains the major therapeutic barrier in ovarian cancer, and current second-line therapies have not proven to be effective [3].

Early detection is needed, so it is essential to understand ovarian cancer initiation as well as what drives its progression. Although some EOC have been suggested to originate from the fallopian tube [4], a majority of reports continue to support the earlier concept that ovarian cancer arises from the ovarian surface epithelium (OSE) [5]. Co-expression of epithelial and mesenchymal markers found in EOC highlights the plasticity of the OSE during epithelial-mesenchymal transition (EMT), and may contribute to neoplastic transformation and acquisition of stemness [6]. Indeed, the identification of ovarian cancer stem and progenitor-like cells (CSCs) [7], ovarian cancer initiating cells (OCIC) [8] and ovarian side-population (SP) cells [9] strongly suggests the involvement of a mechanism like the inherent EMT of OSE cells to confer a phenotypic and genetic plasticity that predisposes them to neoplastic transformation and acquisition of stem cell characteristics.

## Cancer initiation from the ovarian surface epithelium (OSE)

### Normal OSE, epithelial-mesenchymal transition and inclusion cysts

The OSE is a single layer of cuboidal epithelial cells covering the entire surface of the ovary and is responsible for material transport to and from the peritoneal cavity as well as repair of ovulatory rupture [5]. In the 1980s, the first tissue culture systems for OSE from different species [10-14], including human [15,16], were developed. Subsequently, information about the normal functions of OSE expanded rapidly, and established the relationship of OSE with ovarian adenocarcinomas [16,17]; approximately 90% of human ovarian cancers arise from the OSE [13,18-20].

The OSE is considered a primitive type of epithelium and unlike most normal epithelia, expresses both epithelial and mesenchymal markers. Epithelial markers typically expressed include keratins, while vimentin, N-cadherin and MUC1 are the mesenchymal markers that are expressed in the OSE [5]. During the postovulatory repair process and in culture, OSE cells undergo EMT as a part of the wound healing process following ovarian rupture or in response to cell culture conditions [21-24]. Wound healing following ovarian rupture is occasionally accompanied by cell migration across the ruptured surface. The entrapment of OSE in ovarian stroma leads to the formation of inclusion cysts [25] that are thought to represent the site of origin of EOC [5]. In support of this hypothesis, early malignant changes are induced in OSE-lined inclusion cysts, including expression of the EOC marker CA125 in OSE of inclusion cysts but not in normal OSE cells [26-28].

While the normal OSE is protected from the underlying stromal signaling due to separation by a thick cellular layer of the tunica albuginea, inclusion cysts directly expose otherwise naïve OSE to stromal signaling and alter normal gene expression patterns [5]. Such stromal-derived growth factors have been suggested to continue promoting EMT, resulting in neoplastic transformation of OSE within the inclusion cysts [5]. Further, cyst-entrapped OSE may also promote their own transformation through cytokines, growth factors, and other bioactive molecules that accumulate within the confines of inclusion cysts [29,30]. Frequent ovulation contributes to increased EOC risk due to repeated rupture and repair of the OSE. There is convincing evidence that prevention of ovarian rupture, through multiparity or oral contraceptives, is protective against EOC (reviewed in [20]).

## Genetics and epigenetics of cancer initiation and progression

Normal OSE expresses N-cadherin, and little to no E-cadherin. EOCs however, express E-cadherin, especially at the initial phases of transformation; E-cadherin has been shown to induce EMT in OSE cells and is considered an initial step in transformation [16]. E-cadherin expression is reduced in metastasizing cells when cell-cell junctions are abrogated to facilitate cell migration – an adaptation of normal EMT [31]. The phenotypic plasticity of the OSE during disease initiation and progression reflects a flexibility of inherent gene expression that is a pre-requisite for EMT. This feature could be an underlying step facilitating neoplastic transformation, besides accounting for the heterogeneity for expression of epithelial, mesenchymal and 'stemness' genes and exhibit complex patterns of histological differentiation [6,32].

## Ovarian cancer genetics

Comparative genomic hybridization (CGH) of early and late-stage ovarian cancers indicated that chromosome losses are more common than gains in the early-stage tumors, while chromosomal gain and amplifications were mainly confined to the late-stage ovarian tumors [33]. Early EOC may therefore already possess genetic abnormalities that are further propagated in advanced disease states.

Normal OSE, early EOC, and advanced disease are easily classified based on differential gene expression [33-40]. Such array-based findings have been instrumental in understanding the progression of ovarian cancer. Expression profiling of epithelial ovarian cancer of varying histologies has identified genetic signatures that distinguish the major histological types of ovarian carcinoma [41-46].

BRAF and KRAS mutations are common in serous borderline tumors and low-grade serous carcinomas, but very rare in most serous carcinomas of high-grade. Loss of heterozygosity (LOH), microsatellite instability and mutations in PTEN and/or β-catenin genes define endometrioid carcinomas. Clear cell carcinomas are more frequently associated with microsatellite instability [47]. Phosphatidyl inositol 3 kinase (PI3K) and its downstream effector AKT2 have also shown to be amplified in association with a significant proportion of ovarian carcinomas especially the aggressive sub-types [48,49], while the PI3K inhibitor PTEN is mutated in a significant proportion of endometrioid ovarian carcinomas [50]. These findings provide insights into the molecular mechanisms for each clinical phenotype.

EOC expresses a number of genes not found in OSE, including CA125 [5], now an established EOC biomarker [51], folate receptor [52-54] and HE4 [40] that are also overexpressed during disease progression. Upregulation of growth signaling pathways, including Bcr-Abl, Erb-B, Her2/neu, VEGF, and COX-2 are also found in EOC, and contribute to the understanding of EOC progression [55]. microRNA (miRNA) microarrays have also identified a number of over-and-under-expressed miRNA in ovarian cancer; such findings may also become future biomarkers or drug targets [56].

## Cancer epigenetics

A long-standing issue in the field is the sequence of molecular events that lead to epigenetic gene silencing. Clear evidence shows CpG island hypermethylation plays a major role in mediating silencing in cancer and is an early event in cancer development. In some cases, it may even precede the neoplastic process. Because of their heritable nature, hypermethylated CpG islands leave 'molecular footprints' in evolving cancer cells that can be used as molecular markers to reconstruct epigenetic progression during tumorigenesis [57].

As EOC is recognized to be a "methylating" type of cancer; disease progression is strongly linked with silencing of tumor suppressor genes (TSGs) [57,58]. Aberrant epigenetic alterations, represented by loss of DNA methylation leading to global genome hypomethylation as well as gain of promoter-associated CpG methylation, are strongly associated with all stages of tumor formation and progression [57]. Epigenetic remodeling is most obvious at promoter regions of genes that regulate important cell functions [59]. Epigenetic alterations also extend to deviant patterns of histone modifications [60,61] and deregulation of microRNA expression in human epithelial ovarian cancer [62,63]; however, the underlying mechanism and consequences to genome-wide transcriptional changes in cancer are as yet, largely unknown.

Several techniques including methylation-specific PCR (MSP) and bisulfite sequencing are often used to identify temporal acquisition of methylation at individual CpGs within a gene promoter. Chromatin immunoprecipitation (ChIP), using antibodies against epigenetically marked cytosine residues or histones, captures DNA and permits visualization of gene promoters associated with these marks. A temporal theory of epigenetic gene repression has been proposed from data generated from combining MSP and ChIP-chip technologies. This suggests the primary event in transcriptional repression to be chromatin remodeling that in turn, is achieved through histone deacetylation and methylation. Such temporal understanding of CpG island hypermethylation has proved useful for molecular classification of different ovarian cancer types [57], while establishment of a "histone code" of epigenetic marks has provided chromatin signatures that may also be predictive of EOC progression [64-66]. ChIP'ped DNA applied to DNA microarrays (ChIP-chip) is now used to achieve larger-scale epigenetic maps. The intense focus on whole genome-scale epigenetics has spurned the combination of ChIP technology with high-throughput DNA sequencing, yielding ChIP-sequencing (ChIP-seq) technology [67]. Further extension of current bioinformatics analysis software platforms is proving essential for mining and interpreting these massive datasets.

## Cancer stem cells

The recently described cancer stem cell (CSC) hypothesis [68] postulates that tumorigenic potential is limited to a very small subpopulation of cells within the tumor that possess stem-cell properties [69]. The observation that epidermal cancers may arise long after the initial exposure to carcinogen implies that the original carcinogenic event must have occurred in a long-lived stem cell population [70]. These early cancer cells would then give rise to further generations of cells and a resulting tumor mass. CSCs have been identified in several cancers including leukemia [71], brain [72], colon [73], breast [74], and the list is increasing with a fair regularity. The first report of the involvement of stem cells in ovarian cancer came from our lab and described the establishment of an extensive *in vitro *system from the ascites fluid of an epithelial ovarian cancer patient. This comprised of nineteen spontaneously immortalized stem cell clones, each of which is derived from a single cell [6]. Of these, two had tumorigenic and stem cell potential, and could sequentially propagate tumors sequentially in mice over several generations, revealing their identity as ovarian cancer stem cells [7].

Soon after, CSCs were also isolated from ovarian tumors and cell lines by fluorescence-assisted cell sorting (FACS) based on their ability to differentially efflux the DNA-binding dye Hoechst 33342 and expression of the verapamil-sensitive, multi-drug resistance gene BCRP1. This property defines a minute fraction of stem cells termed as the side-population (SP) stem cells [9]. The SP population was further found to be tumorigenic and withstood their identity as CSCs in all the classical stem cell and tumorigenecity assays. More recently, a population of normal murine OSE have been shown to exhibit stem/progenitor cell characteristics, including dye-retention providing evidence for a putative somatic stem/progenitor cells [75]. Another recently described report in ovarian cancer describes the application of expression of stem-cell surface markers CD44 and CD117 (c-kit) to sort out ovarian cancer initiating cells *viz*. OCICs in tumors [8]. The primary ovarian tumor-derived OCIC were capable of serial propagation of the original tumor further establishing them to be essential contributors to tumor growth.

## Similarity to stem cells

Normal stem cells are generally quiescent and possess long-term survival characteristics including self-renewal, DNA repair, and expression of membrane-bound drug-efflux transporter molecules in the cell membrane towards providing resistance to environmental insults. Most, if not all these traits have now been demonstrated in their malignant counterparts [76]. CSCs express a complement of such stem cell markers including Octamer 4 (Oct4), Nanog, nestin, B-lymphoma MMLV insertion region 1 (Bmi-1), stem cell factor 1 (SCF-1) and Notch1, in line with the accepted stem-cell phenotype [7,8].

The first report on the identification of stem cells in ovarian cancer came from our lab. Briefly, we had earlier isolated nineteen single cell clones from the ascites of a patient with advanced serous ovarian carcinoma [7]. While all clones possess stem cell-like characteristics, two of them were found to be tumorigenic and could also grow in an anchorage-independent manner *in vitro *as spheroids. Tumors established from these clones in animal models were similar to those in the human disease in their histopathology and cell architecture. Further, even on serial transplantation, cells of both clones continued to establish tumors. Taken together, the functionality of these clones suggested their identity to be that of cancer stem cells.

## Generation of CSC from transformed stem cells vs. tumor cell dedifferentiation

Although CSCs are being increasingly reported, the exact origin of these cells is still debated. Do CSCs directly originate from normal stem cells, or are they the outcome of ovarian cancer cells that have gained stem cell properties? The former school of thought surmises that normal tissue stem cells give rise to CSCs is supported by the striking degree of similarity between somatic stem cells and cancer cells. As described earlier, this includes the sharing of phenotypic markers as well as the fundamental abilities to self-renew and produce hierarchies of cells at varying levels of differentiation [76-81]. Given these common attributes, it has been proposed that cancers are caused by transforming mutations that occurred in tissue-specific stem cells. This hypothesis is further supported by the fact that among all the cells within a particular tumor, only a small fraction of CSCs are able to regenerate the entire tumor; serial transplantation studies using CSCs and OCIC are able to continue establishing tumors [7,8]. Further studies should provide important insights into the dynamic relationship between developmental plasticity, environmental context and neoplastic transformation [82].

The second school of thought generates the ongoing debate whether tumor growth needs to be driven by CSCs at all [83]. Thus, the alternative hypothesis suggests that reprogramming of cancer cells by dedifferentiation will confer stem-like functions (reviewed in [84]. Tumor cells may also progressively acquire stem cell properties as a consequence of oncogene-induced plasticity [84]. An example in point is that committed myeloid progenitor cells have been shown to acquire leukemia stem cells properties without changing their overall identity, and behave like stem cells through re-activation of genes normally expressed in normal primitive hematopoietic stem cells [85]. It is difficult to attribute the appearance of stem cells in EOC to specifically transformation of normal OSE or to dedifferentiation, but it is considered to reflect anomalous organogenesis and developmental patterning within the transformed tissue [32]. The current definition of a CSC thus, emphasizes its stem cell-like properties, and implies its origin from stem, progenitor or differentiated cells.

We have recently addressed the issue of CSC evolution by profiling the comprehensive model of single cell clones described above as well as several primary tumor samples for mitochondrial (mtDNA) mutations [86]. Divergence of the two cancer stem cell clones expressing a highly mutant mtDNA profile, from normal stem cell clones that express the germline profile, was very distinct. In addition to the two cancer stem cell clones, the mutant group also included 3 non-tumorigenic clones. The latter clones later showed a propensity to undergo transformations. Our findings suggest that stem cell transformation could be the underlying cause of ovarian cancer and that continuing stochastic events of stem and progenitor cell transformation define the increasing aggression that is characteristically associated with the disease.

## EMT and cancer stem cells

Recently, the induction of EMT in immortalized human mammary epithelial cells (HMLEs) were reported to result in the acquisition of not only mesenchymal traits, but also expression of stem-cell markers and formation of mammospheres, a property associated with mammary epithelial stem cells [6]. Furthermore, stem -like cells isolated from HMLE cultures form mammospheres express markers similar to those of HMLEs that have undergone EMT [6]. The intrinsic property of OSE to undergo EMT, gain mesenchymal and stem-cell markers [5] suggests that these cells may have a propensity to re-acquire a stem cell-like phenotype. Further, OCIC, as in the mammospheres, also exhibit classical stem-cell properties [8], suggesting that a similar situation is likely to occur in the context of ovarian cancer.

## Epigenetics of cancer stem cells

Promotion and expansion of stem/progenitor cells predisposes cancer development by expanding a pluripotent population of cells that would otherwise be quiescent or undergo appropriate differentiation [82]. While genetic contributions have been well documented in carcinogenesis, they do not solely explain the origin of most tumors [87]. CSCs harboring similar genetic abnormalities as the parental tumors, are still distinctly different, and may be explained by epigenetic changes [88]. The involvement of widespread epigenetic alterations is now known to occur very early in carcinogenesis even prior to genetic mutations, and suggests such mechanisms to mediate transformation [87]. Alterations include global DNA hypomethylation with localized hypermethylation, and aberrant patterns of histone modifications including atypical lysine acetylation and methylation [60], provide evidence of "epigenetic progenitor model of human cancer" theory [87]. Such early epigenetic events would further evolve during expansion of a progenitor cell pool, followed by hyperplasia and an initiating genetic or epigenetic mutation. Continuing mutagenesis and alterations in chromatin patterns and transient silencing of regulatory genes in stem cells possibly would further facilitate aberrant cell functioning during tumor initiation and progression [89].

Epigenetic modulation of gene expression is also known to be essential for normal function of stem cells. Both normal and malignant embryonic cells generally lack the hypermethylation of DNA found in adult cancers. However, the pattern of epigenetic regulation is disrupted and highly abnormal in cancers, often highlighted by aberrant promoter CpG island hypermethylation and transcriptional silencing of tumor suppressor genes and pro-differentiation factors. Many of the aberrant chromatin modifications are repressive, and act to silence tumor suppressor genes [60] several genes of which normally contribute to differentiation [90]. In normal stem cells, a large majority of such genes are identified in association with a bivalent histone mark in their promoter regions, consisting of a co-occupancy of an activating H3K4me2 mark with a repressive H3K27me3 modification [91,92]. Such a co-existence of activating and repressive marks, in differentiation-control genes, has been suggested to maintain these genes poised in a 'transcription-ready' state, poised for up- or down- regulation [93,94]. The bivalent state of H3K4me2 – H3K27me3 exists in immature stem and progenitor cells as a mechanism of gene regulation, and is more recently identified in adult tumors at a large percentage of genes, revealing a novel chromatin-based mechanism for maintaining pluripotency [92]. The identification of such features in pluripotent embryonal carcinoma cells has led to the realization that bivalent epigenetic marks H3K4me2/H3K9me2 and H3K4me2/H3K9me3 are also additionally associated with CSCs [89].

Ezh2, a polycomb repressive complex (PRC)-dependent histone-lysine methyltransferase is increasingly elevated during prostate cancer progression [95]. During EOC disease progression, DNMT1 expression is elevated [39], is correlated with increased DNA and histone methylation with advanced, chemoresistant ovarian cancer [65,96,97]. Epigenetic regulation of CSCs is thus, a rapidly emerging area wherein relatively knowledge exists at present, but continuing understanding of the normal mechanisms could reveal the aberrant features that mediate and maintain the transformed state, especially in solid malignancies [87,89,98].

## miRNA as targets and/or mediators of epigenetic signaling

MicroRNAs (miRNAs, or miRs) are an abundant class of small noncoding RNAs that function as negative gene regulators. These small, evolutionarily conserved, noncoding RNAs (approximately 20–22 nucleotides) are the result of a complex sequence of processing steps, and mediate critical functions in cell proliferation, apoptosis, and differentiation through regulation of the expression of several critical genes in development and organogenesis (reviewed in [99]. Although a relatively new field, there is already a clear and definitive role for miRNA that function as tumor suppressors were found to be markedly down-regulated in malignant transformation and tumor progression [63]. miRNA has also been shown to directly regulate tumor suppressors and oncogenes in ovarian cancer [62,99-105].

As is well understood, promoter hypermethylation may induce gene silencing. Recently, epigenetics has also been shown to directly impact miRNA expression [106,107]. Furthermore, it is now appreciated that many miRNAs are located within introns of genes, and may be subjected to epigenetic silencing along with the preceding gene [108]. What was once seen as epigenetic downregulation of a single gene has now expanded to include miRNA and the numerous potential downstream pathways of the miRNA targets; for example, the hypermethylation and subsequent repression of let7a-3 in ovarian cancer [109]. DNMTi therapy has been shown to reverse hypermethylation and increase miRNA expression [103], further providing evidence that epigenetics can directly silence miRNA. miRNA gene silencing in stem cells has been shown to be mediated by Polycomb group proteins, resulting in tissue-specific expression in differentiated cells [110], revealing an epigenetic-mediated regulation of miRNA as yet another regulatory mechanism that influences stemness and cell differentiation. However, the regulation of miRNAs at the transcriptional level remains relatively unexplored.

The use of miRNA microarrays are proving useful in delineating the complex miRNA regulatory networks. In combination with gene expression and ChIP arrays, such miRNA analyses will allow correlations to be made between epigenetic marks and subsequent changes in gene expression, miRNA expression, and impact on downstream signaling pathways [56,62,63,111-113]. Furthermore, miRNA may join the ranks of biomarkers; expression of the miRNA-200 family has been shown to define the epithelial ovarian cancer phenotype [114,115]. Therefore, transcriptional, epigenetic and now miRNA-mediated regulatory mechanisms all appear to coordinate the molecular mechanisms driving pluripotency and self-renewal in ovarian cancer [116].

## Targeted therapy to advanced disease and cancer stem cells

The rate of mortality in ovarian cancer has changed little in the past three decades [1,2]; drug resistance remains the major therapeutic barrier [3]. Early detection is critical [117], and many genes that are specifically overexpressed in the context of ovarian cancer provide potential biomarkers for ovarian cancer detection [33-37,118]. Changes in gene expression of these biomarkers may also be used as surrogate tests for chemotherapy response, such as CA125, osteopontin, MUC1, and HE4 [40,51]. Although chemotherapeutics target rapidly proliferating tumor cells and provide temporary remission, only the bulk of tumor cells is destroyed, and drug-resistant stem cells remain. In line with clinical observations, it is currently believed that recurrent disease is repopulated by these chemoresistant CSCs [119] that retain their drug-resistant phenotype, proliferate, pass along drug resistance to their progeny and thus repopulate a tumor that is fully refractory to further treatment [1,3]. Indeed, such cells from solid tumors have been directly demonstrated as being chemo- and radioresistant, with a potential role in disease recurrence [120-122]. CSCs and their relevance for tumor progression and tumor therapy have been extensively reviewed [123].

## Epigenetics-based gene therapy

One therapeutic approach for targeting chemoresistance may be to reverse the epigenetic marks in chemoresistant cells. Small interfering RNA (siRNA) raised against DNMT1 has been shown to restore estrogen receptor-alpha (ERα) signaling in ERα-negative human breast cancer cell lines, thus enabling the use of antiestrogens as therapy [124] while overexpression of a dominant-negative histone H3 lysine 27 mutant (H3K27R) de-repressed epigenetically silenced tumor suppressor genes and reversed drug-resistance in ovarian cancer cells [65]. Such studies provide a proof-of-concept that epigenetic alteration can directly impact ovarian cancer chemosensitivity. Several chemical inhibitors of epigenetic enzymes, targeting DNMT and histone deacetylases (HDAC), have shown promising anti-tumorigenic effects for some malignancies (reviewed in [125,126]. Treatment of cancer cells with the DNMT inhibitors 5-aza-dC, decitabine and zebularine show promise in reversal of repressive histone mark patterns and resensitization of ovarian cancer cells to chemotherapy [127,128], and several are currently in clinical trials [125,126].

Inhibitors of class I histone deacetylases (HDACi) have been shown to suppress ovarian cancer cell growth and provide an option for clinical use [129]. HDACi are divided into four groups: short-chain fatty acids, hydroxamic acids, cyclic tetrapeptides, and benzamides. The small-chain fatty acids butyrate, and valproic acid (VPA) (originally regarded as anti-epileptic drug), were the first known HDAC inhibitors [130,131]. Although not exceedingly specific, these compounds laid the foundation of HDACi and are tools for studying the structure and mechanism of HDACi. Newer HDACi, like suberoylanilide hydroxamic acid (SAHA), have been rationally designed with high affinity for the zinc ion within the HDAC catalytic domain [132]. The HDACi depsipeptide has the capability to activate silenced genes by decreasing both CpG and H3K9 methylation at gene promoters, suggesting HDAC inhibition induces additional chromatin regulation aside from histone acetylation [133]. Indeed, it has been established that combinations of DNA methylation and HDACi are more potent for gene re-expression than either alone [97,134]. VPA, SAHA, depsipeptide, and other HDACi are currently in clinical trials, alone and in combinations with DNMTi (reviewed in [125,126]). Combinatorial epigenetic therapy may also be useful in targeting stem cells; DNMT1 gene knockout combined with HDACi has been shown to be lethal to embryonic stem cells [135]. Such therapy may therefore extended to targeting against cancer stem cells. One potential drawback of epigenetic therapy is the possibility that these agents can inhibit or reverse normal developmental processes or accelerate cellular differentiation and tissue ageing [136-138]. This may not be surprising since most HDACi and DNMTi compounds were originally discovered as differentiating agents [126,139-141], and must be considered alongside efficacy.

## miRNA and siRNA therapy

siRNA have been employed as transcriptional inhibitors of oncogene and growth factor signaling. Oct4 plays a key role in the maintenance of pluripotency and proliferation potential of stem cells, and siRNA directed against OCT4 induces cell apoptosis in stem cells [142]. Synthetic miRNA have been utilized to target glioma-associated antigen 1 transcription factor, and induce apoptosis in pancreatic tumor cells, providing an alternative to siRNA-mediated gene silencing [143]. The concept of siRNA and miRNA-mediated gene silencing is solid, but is limited clinically by methods of delivery [144].

miRNA expression is correlated with various human cancers and indicates that deregulated miRNAs can function as classical tumor suppressors and oncogenes [145,146]. miRNAs have been shown to repress the expression of important cancer-related genes and might prove useful in cancer diagnosis and therapy. For example, let-7 miRNAs has been found to be downregulated in different types of cancer, suggesting that it acts as a tumor suppressor genes [146]. The oncogene RAS is one mRNA transcript targeted by miRNA let-7, revealing a possible mechanism for let-7-mediated tumor suppression [147]. Several groups have also described the deregulated expression of miRNAs in cancer by microarray analysis. miRNAs expression profiling can distinguish between different types of cancers and even between different subtypes of tumors from the same cancer type [148-150].

An individual miRNA may have dozens or even hundreds or transcriptional targets [99]. Based on this premise, miRNA-targeted therapies have introduced the revolutionary therapeutic concept of 'one hit, multiple targets' [151]. Therapeutic interventions for miRNAs could be used to 'correct' the miRNA expression levels and, consequently, 'normalize' the expression of their numerous mRNA targets in cells, some of which may be encoded by oncogenes and tumor suppressor genes [151]. An important chicken-or-egg question remains: Are miRNAs differentially expressed as a consequence of the cancer state, or does cancer cause the deregulated expression of miRNAs? Both scenarios are likely true; miRNA have been shown to be epigenetically silenced [106-108], and miRNA have been shown to modulate expression of epigenetic machinery in human cancers [152,153].

## Conclusion

The inadequacy of standard therapies is currently being considered as a failure of existing chemotherapeutics to target ovarian CSCs resulting in inevitable relapse [119,154]. As CSCs are believed to be responsible for perpetuating recurrent and chemorefractory disease[119], a great need exists for therapies that target the small percentage of tumorigenic progenitors and may provide a much needed breakthrough in treatment of advanced cancer and chemoresistant disease [119]. Integrating genomic, epigenomic, and miRNA microarray technologies have begun to reveal a coordinated network of epigenetic-mediated regulation of gene and miRNA expression which promotes the stem cell phenotype [110]. We can soon expect an explosion in the volume of epigenetic data available as continued advances in array-based whole-genome/epigenome analysis more clearly define the genome and epigenome of cancer and CSCs. Cancer epigenetics research has led to the field of translational epigenetics, a growing range of epigenetic inhibitors, and raised intense interest in the development of additional epigenetic drugs with greater specificity and efficacy in clinical settings. Great potential lies in the development of novel epigenetic-based therapies targeting DNA methyltransferases and histone deacetylases [155].

The development of novel therapeutics requires the continuing application of existing technologies in genomics, proteomics and bioinformatics to resolve the complex relationships involved. Continued investigation of genetics, epigenetics, and the mechanisms responsible for the initiation and progression of ovarian cancer will allow for further understanding of the relationship between stem cell-based tumorigenesis and epigenomic alterations. Further characterization of tumorigenic cell populations will identify molecules expressed in CSCs that could then serve as targets. With the identification of definitive targets, this fraction of cancer cells that can rapidly develop the critical tumor cell mass could be eliminated. Consequently, defining the unique properties of ovarian CSCs remains a high priority for developing early diagnostic and effective therapeutic strategies against ovarian cancer.

## Abbreviations

5-aza-dC: 5-aza-deoxycytidine; Bmi-1: B-lymphoma MMLV insertion region 1; CGH: comparative genomic hybridization; ChIP: chromatin immunoprecipitation; ChIP-seq: high-throughput ChIP-sequencing; CIC: cancer-initiating cell; CpGi: CpG island; CSC: cancer stem cell; DNMT: DNA methyltransferase; DNMTi: DNA methyltransferase inhibitor; DMH: differential methylation hybridization; EMT: epithelial-mesenchymal transition; EZH2: Enhancer of Zeste Homolog 2; FACS: fluorescent-assisted cell sorting; HDAC: histone deacetylase; HDACi: histone deacetylase inhibitor; HMLEs: immortalized human mammary epithelial cells; LOH: loss of heterozygosity; miRNA: microRNA; MSP: methylation-specific PCR; OCIC: ovarian cancer-initiating cell; OCT4: Octamer 4; OSE: ovarian surface epithelium; PRC: polycomb repressive complex; SCF-1: stem cell factor-1; siRNA: small interfering RNA; TSG: tumor suppressor gene.

## Competing interests

The authors declare that they have no competing interests.

## Authors' contributions

NB drafted the manuscript. SAB conceptualized, edited and revised the manuscript. All authors have read and approved the final manuscript.
